# The Card Sort Task for Self-harm (CaTS): a review of the development, application, and adaptation of a novel tool for the assessment and contextual understanding of self-harm

**DOI:** 10.1186/s12888-026-08377-3

**Published:** 2026-07-17

**Authors:** Emma Nielsen, Katherine Bird, A. Jess. Williams, Joanna Lockwood, David Jobes, Amy Brausch, Ruth Wadman, Delfina Bilello, Camilla M. Babbage, Sarah Cassidy, Laura del Carpio, Blandine French, Carol MacDonald, Victoria Newell, Mirabel Pelton, Susan Rasmussen, Ellen Townsend

**Affiliations:** 1 National Institute for Health and Care Research (NIHR) Greater Manchester Patient Safety Research Collaboration (GM PSRC), University of Manchester, Manchester, UK; 2 Self-Harm Research Group, University of Nottingham, Nottingham, UK; 3 National Centre for Suicide Prevention and Self-Harm Research, Population Data Science, Swansea University Medical School, Swansea, UK; 4 Department of Informatics, Bush House, Strand Campus, King’s College London, London, UK; 5 Institute of Mental Health, Jubilee Park, University of Nottingham, Nottingham, UK; 6 Department of Psychology, The Catholic University of America, Washington, DC USA; 7 Department of Psychological Sciences, Western Kentucky University, Bowling Green, KY USA; 8 Department of Health Sciences, University of York, York, UK; 9 Department of Experimental Psychology, University of Oxford, Oxford, UK; 10 NIHR MindTech MedTech Co-Operative, Institute of Mental Health, Mental Health & Clinical Neurosciences, University of Nottingham, Nottingham, NG7 2TU UK; 11 School of Psychology, University of Nottingham, Nottingham, UK; 12 Health Technology Assessment in Primary Care and Mental Health (PRISMA Group), Fundació Sant Joan de Déu, Barcelona, Spain; 13 Hearing Sciences, Mental Health and Clinical Neurosciences, School of Medicine, Faculty of Medicine & Health Sciences, University of Nottingham, Nottingham, UK; 14 NIHR Nottingham Biomedical Research Centre-Hearing, Nottingham, Nottingham, UK; 15 School of Psychology, University of Sheffield, Sheffield, UK; 16 Autism Research Centre, Department of Psychiatry, University of Cambridge, Cambridge, UK; 17 Department of Psychological Sciences and Health, University of Strathclyde, Glasgow, UK

**Keywords:** Self-harm, Assessment, Methodology, Non-suicidal self-injury

## Abstract

Developing clear, adaptable, and engaging methodologies is critical for advancing the understanding and prevention of self-harm and suicide. This paper presents a review of the development, application and adaptation of the Card Sort Task for Self-Harm (CaTS), a structured, interactive tool designed to map individual pathways to self-harm. Grounded in psychological theory and co-developed with input from people with lived experience, CaTS uses 117 evidence-informed cards representing thoughts, feelings, events, behaviours, and support-related factors. Participants place these cards along a timeline spanning the period before and after a self-harm episode, enabling the identification of both risk and protective factors as well as the sequencing of relevant experiences. The inclusion of blank cards supports personalisation, ensuring the tool remains sensitive to diverse experiences. Unlike traditional psychometric measures, CaTS emphasises holistic understanding and temporal patterning. Since its initial application, CaTS has been adapted for use with varied populations, delivery formats, and research settings. This paper outlines the conception and evolution of CaTS, highlighting the tool’s potential for clinical, research and participatory applications, and utility in training and public engagement. By offering a flexible, person-centred approach, CaTS advances inclusive and nuanced exploration of self-harm processes, offering a novel way to explore often-overlooked, complex temporal dynamics.

## Clinical trial number

Not applicable.

## Background

Self-harm, defined as any self-injury or self-poisoning, regardless of intent [1, 2] is a multifaceted [3], prevalent [4] and significant [5, 6] public health concern, resulting from complex temporal interactions between biological, social, emotional, and psychological factors [7, 8]. Experiencing self-harm thoughts and/or self-harming can be distressing; self-harm is also associated with multiple negative health, social, and wellbeing determinants and outcomes (e.g. [9–13]). Concerningly, self-harm is also the most significant and reliable predictor of death by suicide [14, 15]. Indeed, a large-scale study found suicide risk was 49 times greater - compared to the general population - in the year following self-harm [15]. In 2019, approximately 703,000 people died globally by suicide [16]. Self-harm reduction efforts are a vital facet of effective suicide prevention strategies [2] and hold promise in improving wider health and wellbeing outcomes.

Multiple risk factors typically precede self-harm, for example, psychological factors such as emotional dysregulation [17, 18] and hopelessness [10, 19], and significant life events, particularly relationship difficulties [20]. Little is understood about temporal interactions and the dynamic interplay between multiple factors experienced by people who self-harm. Self-harm assessment measures and risk tools usually restrict focus to isolated factors [3, 21]. The oversimplification of multifaceted phenomena, ignoring interactive and temporal dynamics is of limited benefit in furthering the academic understanding of self-harm [22] and holds limited to no clinical utility (in assessment and in understanding risk) [23, 24]. Self-harm is complex. Understanding someone’s experience is more complicated than simply considering the presence, absence or even additive nature of factors. Experiences are not static or linear and requires rich information for meaningful exploration; it is conceptually limiting to try and capture non-concrete constructs, for example, multifaceted psychological states, in isolated factors. Indeed, research to better understand temporal relationships between self-harm factors is recommended by researchers in the suicide-prevention field [25, 26].

To address the distinct needs inherent to self-harm, the Card Sort Task for Self-Harm (CaTS) was developed [3]. CaTS affords the opportunity to capture the complexity and temporal dynamics of self-harm. The card-based tool blends quantitative and qualitative facets enabling the identification of key or co-occurring antecedents to an episode of self-harm, organised in temporal order and sequiturs, across a timeline. Identifying significant antecedent-sequitur transitions in the pathway to self-harm could help identify and prioritise targets for intervention and develop appropriate support and interventions [27–29]. In this paper, we first introduce CaTS as a unique and flexible research assessment tool. We then summarise the evidence supporting the development and application of this unique tool beyond its inception, in research, training, public engagement activities and clinical practice. Finally, we will discuss potential future uses and directions for CaTS.

## Card Sort Task for Self-Harm (CaTS)

CaTS is a structured, card-based tool. CaTS uses 117 cards, labelling thoughts, feelings, events, behaviours, and support/service-related items which have been associated with self-harm. CaTS was developed to simultaneously examine multiple factors leading up to and following an episode of self-harm. Participants create their self-harm pathway by placing cards relevant to their self-harm underneath seven timepoints (“*6 months before”, “1 month before”, “1 week before”, “1 day before”, “1 hour before”, “self-harm”, “afterwards”*). This enables CaTS to examine distal and proximal self-harm risk and support factors and the temporal ordering of these factors.

The original card deck (see https://selfharmresearchgroup.info/ for access) was developed using robust risk factors and longitudinal evidence (e.g. [30, 31]), theory and model [32–34], and was reviewed by a multidisciplinary team of researchers, clinicians, and young people with lived experience of self-harm (see Sect. 2.1 for discussion). This approach ensured multiple perspectives fed into the cards included, resulting in a comprehensive set of cards relevant to young people, clinicians, and researchers. A paucity of research limits our understanding of protective factors in self-harm. Access to informal [35, 36] and formal [37] support can help (although not always [37, 38]). CaTS included cards pertaining to support and service factors to further explore this. There are also blank cards available to use in the card sort. This allows participants to add factors unique to their pathway or that are not already captured within the card deck.

**Fig. 1:**
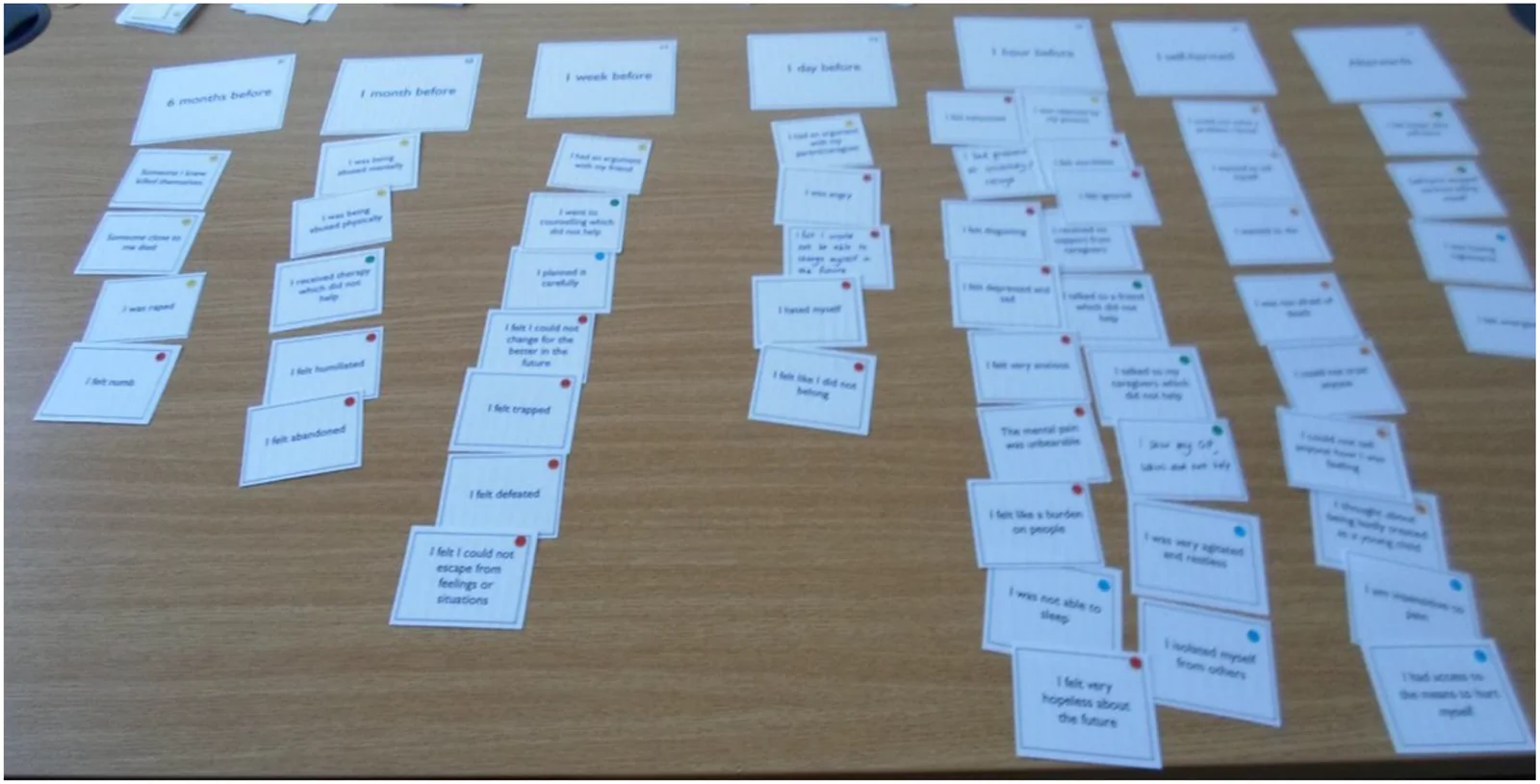
An example CaTS card sort, with relevant cards placed (vertically), in a sequence, under timeline cards (which run horizontally at the top of the image)

CaTS was initially designed as a paper-based research tool, to support and scaffold the generation of a timeline of experiences relating to an episode of self-harm (See Fig. 1). In its original format, CaTS was used within an in-person setting, facilitated by a researcher.

CaTS is not a psychometric assessment measure, but rather a tool to capture the individual-specific, subjective experience of each person completing the task. The original aim of the task was to begin to unpack the often-overlooked complexities of self-harm and to determine whether self-harm exhibited a sequential pattern [3]. Building on the work of Bakeman and Gottman (1997 [39]) we used lag sequential analysis examine temporal statistical dependencies between events. This can be applied to CaTS to identify characteristic signatures in the data, and key transitions that may enable identification of early warning signs of self-harm.

CaTS shares similarities with chain analysis, a form of functional behavioural analysis used in Dialectical Behaviour Therapy (DBT). Chain analysis helps individuals identify sequences of thoughts, feelings and behaviours in order to assess and modify unhelpful responses to distress, such as self-harm [40]. However, while chain analysis in DBT focuses on an individual and their environment, the use of specific time points in CaTS (from *“6 months before”*, to *“afterwards”*) permits a method that allows a systematic description and comparison of significant sequences related to self-harm across individuals. Alternative timeline methods are used in suicide research, such as the Timeline Follow-Back Interview (TLFB-SA [41–42]): (which typically focuses on the 48 hours prior to an attempt), the Pathway to Suicidal Action Interview (PSAI [43]): (which requires an individual to narrate their experience of a suicide attempt with follow-up questions), and Ecological Momentary Assessment (EMA) (which typically involves intensive real-time, real-world assessment of a person current circumstances, over a duration of days to weeks). CaTS is distinct from these methods, and offers advantages, since: [1] minimal training is required, [2] CaTS uses a wider timeline to capture proximal and distal factors, and [3] CaTS does not require verbalisation for completion.

### Patient and public involvement (PPI) in the development of CaTS

**Fig. 2:**
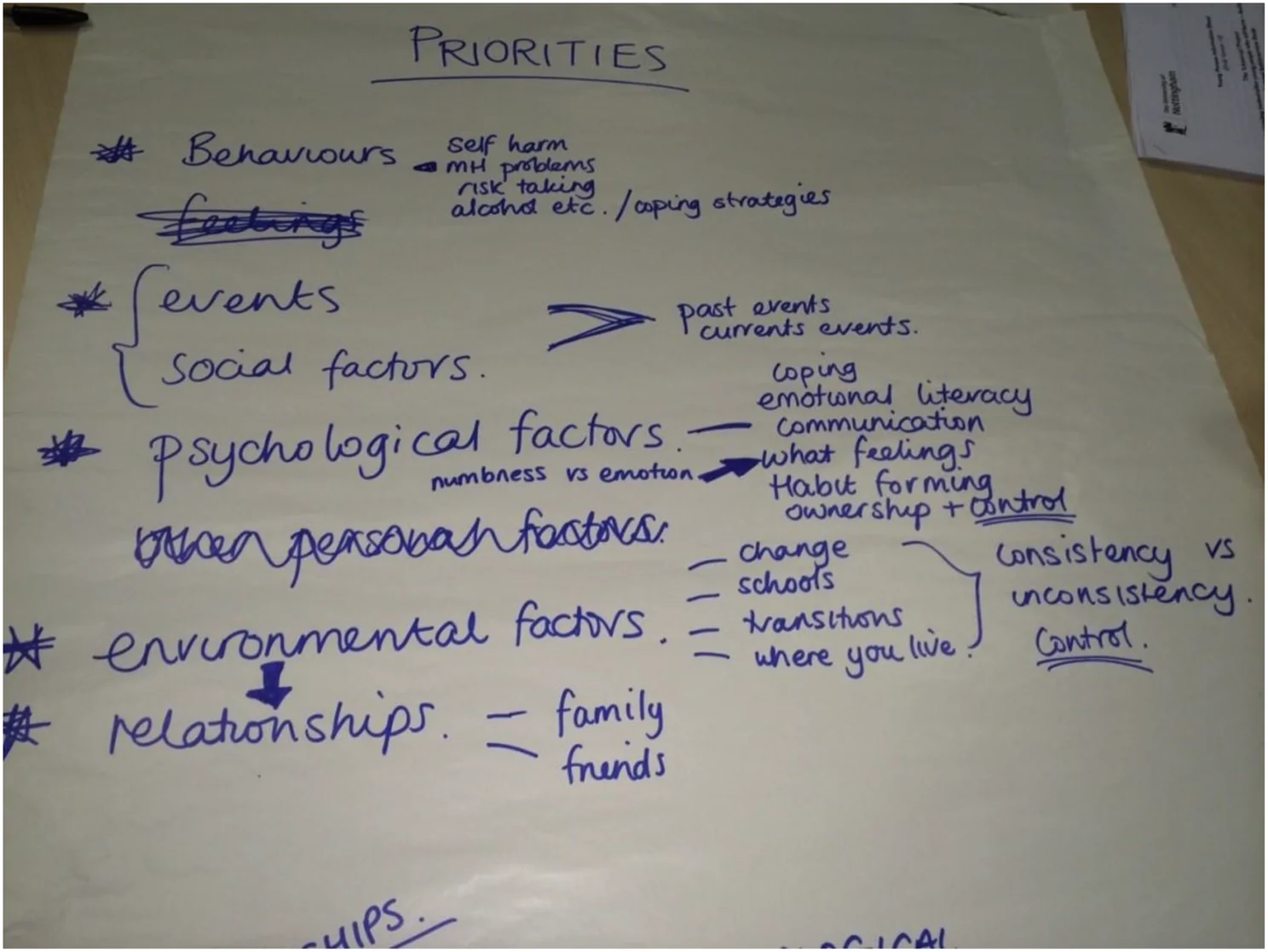
Priority settings activities within our YPAG

**Fig. 3:**
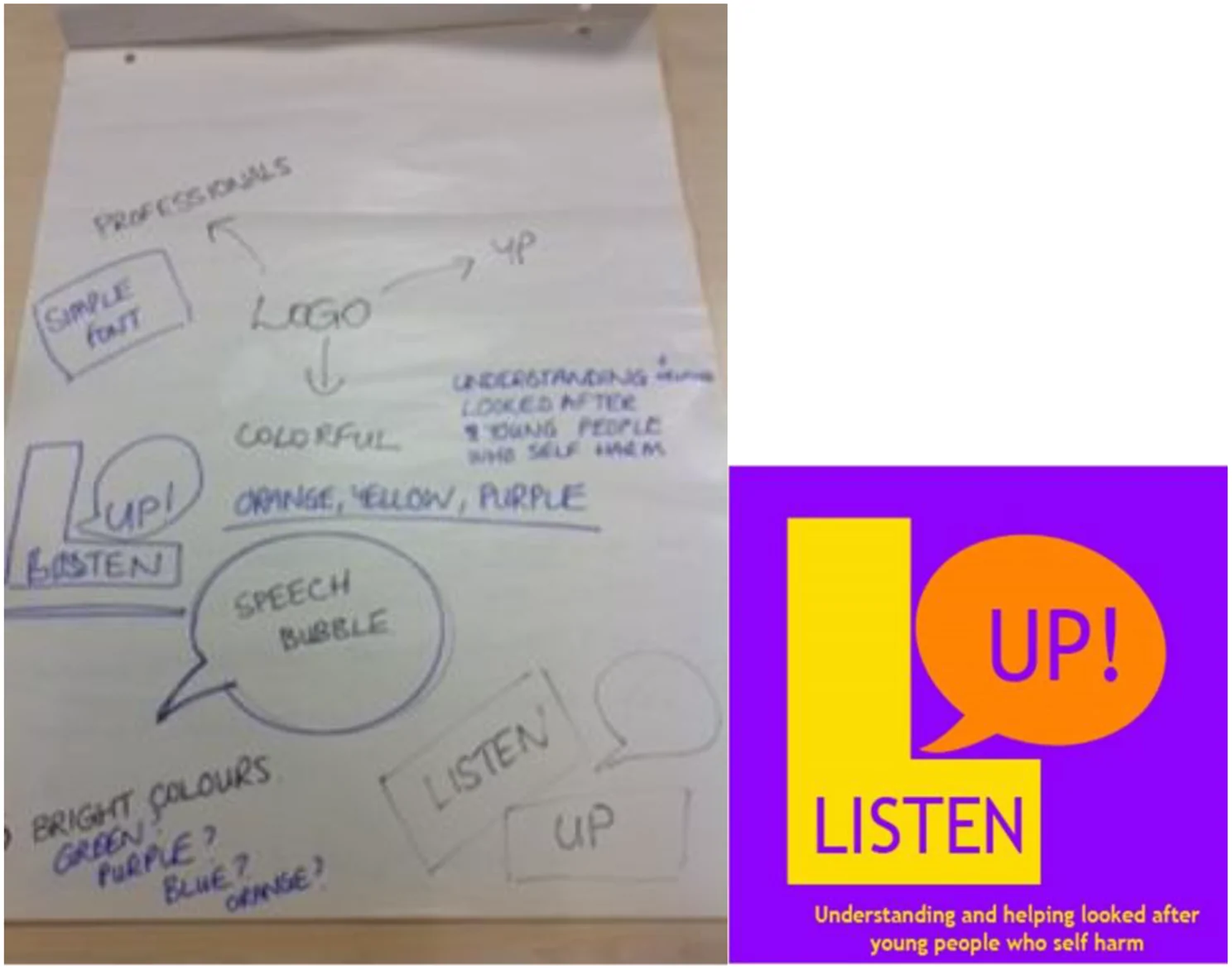
Developing the project branding within our YPAG

The involvement of people with lived experience is vital in creating a novel research tool, especially one that covers a sensitive topic such as self-harm [44]. Involvement has been shown to improve research design and delivery [45] and is associated with improved acceptability [46]. During the initial development, testing and dissemination of the CaTS, the research team worked closely with young people with lived experience of self-harm (this research and engagement work was funded by an NIHR grant - the ‘Listen-up! Project [Award ID: 023/0164]). The project recruited a Young Person’s Advisory Group (YPAG), which was supported by, and run at, a local user-led support service for self-harm. Young people were involved throughout the research lifecycle from setting priorities (see Fig. 2) to assisting in dissemination. This YPAG added items to the card deck which they felt were important, but missing (for example, *“I moved to a different school”*). The YPAG also developed the study branding (See Fig. 3), advised on advertising and recruitment practices, assisted in interpretation, and identified priorities for future research and iterations of CaTS. The YPAG also made suggestions about mood repair tasksand how the research team could ensure participants exited the study on a positive note.

Involvement remains a central ethos across CaTS work. Further developments of CaTS for specific groups – for example, with autistic people - have involved working in partnership with those groups to ensure that the instructions for the task are clear, the timeline and cards are relevant and clear, and CaTS method is acceptable and usable to the target group. Key stakeholders are not just those with lived experience. The team’s involvement activities have been expanded to include formal (for example, education and healthcare professionals) and informal supporters (for example, parents and carers). This work is noted throughout the latter half of this paper.

### The initial application of CaTS

The original CaTS was used within the Listen-Up! Project [3, 47], which explored the experiences of the first and most recent episode of self-harm among looked-after young people (i.e., in the state social care system) and non-looked-after young people. The young people were instructed to go through the timeline chronologically leading up to the self-harm event. Cards could be placed only once in the timeline. CaTS generally took between 20 and 22 minutes to complete [47], with an average of 27 cards (range: 2–59) being used to describe the initial episode of self-harm and 29 (range: 4–63) for the most recent [3]. We demonstrated that self-harm does follow a sequential pattern and identified that the important transitions uncovered in our analyses could help us find potential intervention points and targets [3].

### More recent evidence for the utility of the CaTS

CaTS was quickly recognised – by involved young people, research participants, and professionals working to support young people who self-harm – as an innovative tool to better understand experiences of self-harm. Therefore, further research across a range of contexts and domains began to use the tool (see Table 1 for summary). In practice, these are not distinct categories and both group identities and learnings intercept and overlap. Broadly, research to date has explored using CaTS with: [i] young people, [ii] informal supporters, and [iii] those in at-risk, yet often under-researched and under-served, populations for self-harm, including neurodivergent people. Here, we will briefly highlight these projects but encourage readers to refer to the original manuscripts for full details.

CaTS has been used across age populations: young people, working-age adults, and older adults. In the United States, CaTS has been adapted for use with distinct forms of self-harm. In samples of both adolescents (ages 12–17) and young adults (ages 18–25), CaTS has been used as a tool to understand pathways to recent suicide attempts and recent non-suicidal self-injury (48, in prep).

#### Young people

CaTS was initially designed for use with young people between the ages of 11–21 years [3, 47]. This has since been expanded to young adults aged to 25 years (e.g. [48]), (as well as adults). Since the initial application, researchers have furthered adapted the task to examine key transitions in more detail [49, 50]. Lockwood and colleagues [49] found, in terms of exploring this challenging topic with young people, that the ability to visually and physically review and manoeuvre cards, creating patterns between items, appeared to help young people pinpoint and describe salient factors while clarifying personal understanding, and facilitated the controlled exploration of complex phenomena. This supports the research utility of card-sort methods for self-harm in youth within qualitative work and echoes earlier enthusiasm and informal feedback received during the Listen Up! Project [3].

Lockwood and colleagues [50] also compared the pathway of factors leading to self-harm in young people and adults using an online version of CaTS and found common risk factors (such as *“acting on the spur of the moment”*) consistent at first ever and most recent self-harm across ages, but also characteristic differences, such as burdensomeness being a more notable marker in adult pathways. This highlights the fluid nature of risk factors, and the importance that research tools are capable of capturing this fluidity.

#### Informal supporters

CaTS has been adapted to consider the experiences of informal supporters, such as friends (CaTS-For Friends/CaTS-FF [51]); and parents or carers of young people who self-harm (CaTS-parent [52]). In a retrospective qualitative study, Bilello and colleagues [51] interviewed friend supporters to consider the role and experiences of friends in adolescent self-harm. The CaTS-FF included new cards to focus on friendship-related factors (e.g., “*My friend was rejected by their friends/peers*”, “*My friend was trying to push people away*”), and adjusted the timeline to ensure the positionality of the task to the supporter (e.g., “*My friend*”). Friends were asked to sort the cards according to their knowledge or perception of the factors contributing to their friends’ self-harm. Used within a qualitative framework, the CaTS-FF served both as a visual tool for representing friends’ perceptions and understanding of adolescent self-harm, while also acting as a scaffold for exploring their own experiences, perceptions and responses to self-harm and how these evolved alongside their friends’. This allowed identification of the friends’ evolving role throughout the experience, and the impact self-harm had on their own wellbeing and friendship over time. CaTS was also used in a retrospective qualitative study to explore the role of friendships in self-harm experiences of young adults who self-harmed during adolescence (CaTS About Friends/ CaTS-AF [53]). To align with the aims of the research, the original CaTS was adapted to include cards with a greater focus on friendship-related factors, protective factors and relational dynamics (e.g., “*I felt accepted/I felt I belonged*”, “*I discussed self-harm with my friends*”). This approach allowed exploration of relevant peer processes, including informal friend support and peer influence on self-harm. CaTS configurations provided a shared understanding of the contextual factors contributing to participants’ self-harm and served as prompts for scaffolding semi-structured interviews. During these interviews, cards relevant to the primary research questions were discussed and collaboratively unpacked. This format enabled an in-depth exploration of specific aspects related to self-harm (i.e., friendships), whilst also preserving participant-led narratives which highlighted the most meaningful factors for individuals’ experiences.

These adaptations hold additional value in developing in-depth understanding of self-harm experiences, such as exploring the wider impact of self-harm behaviour, considering informal supporting or contagion. Lockwood and colleagues [52] considered the potential of CaTS as a tool targeted at parent-carers. Using a familiarisation video and an example online format of CaTS on a digital whiteboard, parents were guided through CaTS and asked to provide feedback on the suitability and value of the tool to enhance parent/carer understanding of self-harm in young people. Parents were asked to complete CaTS by considering their child’s self-harm experiences or a vignette, and to consider to what extent the tool would support parent-led conversations about self-harm (e.g., with their child, another family member, practitioner). In these settings, CaTS allows informal supporters to explore and share their experiences of self-harm in relation to their loved one or an example child, while being removed enough to evaluate these experiences and consider the utility of this tool.

#### At-risk, yet often under-researched and under-served, populations

Recent research has been conducted utilising CaTS with at-risk yet often under-researched and under-served populations, including LGBTQIA+ people [54], autistic people (CaTS-Autism, CaTS-A: 56 Pelton et al., in prep), those with co-occurring health conditions [55] and bereaved young people [56]. These projects have considered specific adaptations and additions relevant to at-risk populations, taking into account those with different communication needs. For example, autistic people often find assessment tools developed for the general population to be unclear and not relevant to their experiences [57], and there are currently no measures validated to assess self-harm in this population [58]. The flexible structure of CaTS may therefore be preferable, allowing for non-speaking contributions or accommodating those who have difficulty recognising and describing their emotions (alexithymia [59]). Moreover, CaTS gives autistic people the space to share unique autism-specific experiences of self-harm, that may relate to sensory processing differences or stimming (self-stimulation) [60, 61], that would not be captured otherwise [62].

In other iterations, CaTS has been adapted as a means of identifying key variables and transdiagnostic features of self-injurious behaviours, suicidal thoughts and tinnitus via interviews with adults with experience of both phenomena. The card sort process was used to elicit the significance, importance, salience, sequence, and interactions between elements of each participant’s experiences to develop a transdiagnostic model. Participants were invited to make their own cards to reflect how they cope and the support they have found helpful. Thirteen out of fourteen participants found CaTS to be a positive process which helped them to reflect upon, understand and validate their experiences and recovery [55].

CaTS was used as part of a mixed-methods study exploring the bereavement experiences of young people aged 16–18 (*n* = 13) who had lost someone important to them to different causes of death [56]. A subsample of participants who reported experience of self-harm thoughts or behaviours were invited to complete CaTS following interview. In line with previous recommendations [47], they also rated their confidence in recalling these experiences, with more recent episodes of self-harm remembered with greater confidence. CaTS proved useful as a structured way to explore these complex experiences, allowing participants to engage with the researcher to the extent they felt comfortable, or to complete the task more independently if they preferred.

### Acceptability

The Theoretical Framework of Acceptability (TFA [63]); understands acceptability as a composite of seven domains, spanning affective attitude, burden, perceived effectiveness, ethicality, intervention coherence, opportunity costs and self-efficacy. While the acceptability of CaTS has not been systematically explored in a standardised approach, we have assessed each domain of the TFA within our research work to date. For example, careful consideration has been given to the number of cards within the CaTS deck to strike a balance between sufficient scope to capture complex phenomena while remaining manageable and not overly time consuming to complete. This is aligned with considerations under the burden domain of the TFA. In partnership with researchers, the task has been developed and evaluated from lived experience perspectives, furthering acceptability and aligned ethicality.

Participants receive CaTS well. Across studies, visual analogue scales (VAS) asking about mood both before and after completing both online and in-person CaTS studies have demonstrated that completing CaTS does not negatively impact participants’ mood [3, 50, 54, 56, 64]. One study by Lockwood and colleagues [50] found that young adults did report a marginally significant reduction in mood compared to adults. This may relate to their self-harm being more recent. However, other CaTS studies in the same age group (<25-years) do not share these findings. Instead, VAS data across studies finds CaTS an acceptable research tool, which does not negatively impact participants’ mood. Furthermore, qualitative studies informally captured similarly positive feedback [49, 51, 54]. Bilello et al. [51] sent a follow-up email two-weeks after interviews took place prompting participants to share thoughts or comments about CaTS. Bird et al. [54] captured similar verbal feedback regarding CaTS’ acceptability and possible cathartic impact on participant mood. Responses across all studies emphasised the benefits of participation. While this data did not form part of the analysis or final papers for either study, they support VAS findings from existing CaTS literature, which indicates taking part in research utilising CaTS does not negatively impact participants and may even provide some personal benefit. This aligns with the affective attitude and opportunity cost facets of acceptability. Furthermore, participants felt CaTS was a helpful research tool which may help participants contextualise their self-harm (e.g. [3, 54]), or facilitate understanding of the several factors encompassing others’ self-harm experience (e.g. [51]). Studies that adapted CaTS for suicide attempts and non-suicidal self-injury in both adolescents and young adults also collected participant feedback on the task. Overall, participants rated CaTS positively in terms of appropriateness, usefulness for understanding their own self-harm, ease of use, and feasibility and helpfulness of using CaTS in therapy [65].

As with many alternate self-harm assessment tools, CaTS relies on individuals’ retrospective accounts of their self-harm journey. As with other retrospective methods, recall bias may influence if events are remembered, how they are remembered and how their sequence is constructed, including inaccuracies in the timing and sequencing of reported events [66]. However, rather than simply aiming to establish an objective chronology, CaTS is designed to capture participants’ subjective interpretations of their experiences over time. CaTS has been used with a variety of populations, in paradigms with a researcher present to support engagement with the task and online, with no live researcher support. Participants have been consistently able to complete a card sort, without significant noted concerns regarding difficulty remembering experiences or responses that occurred weeks or months before on the timeline. Across extensive involvement work, lived experience advisors have consistently reported that the task is acceptable. Taken together, we are confident that CaTS is a valuable method for illustrating how individuals make meaning of their self-harm trajectories, including which events are perceived as significant and why. These accounts therefore provide insight not only into past events, but into participants’ current understandings of their self-harm experiences.

The latter half of this paper will explore key developments and implementations of CaTS, focusing on the diverse delivery formats utilised. We will also examine the broader impact and potential beyond academia, including applications in training, clinical practice, and public engagement strategies. Key learnings to date will be highlighted, with suggestions for future advancements.

## Implementation

### Delivery of CaTS: offline, online, and app

**Fig. 4:**
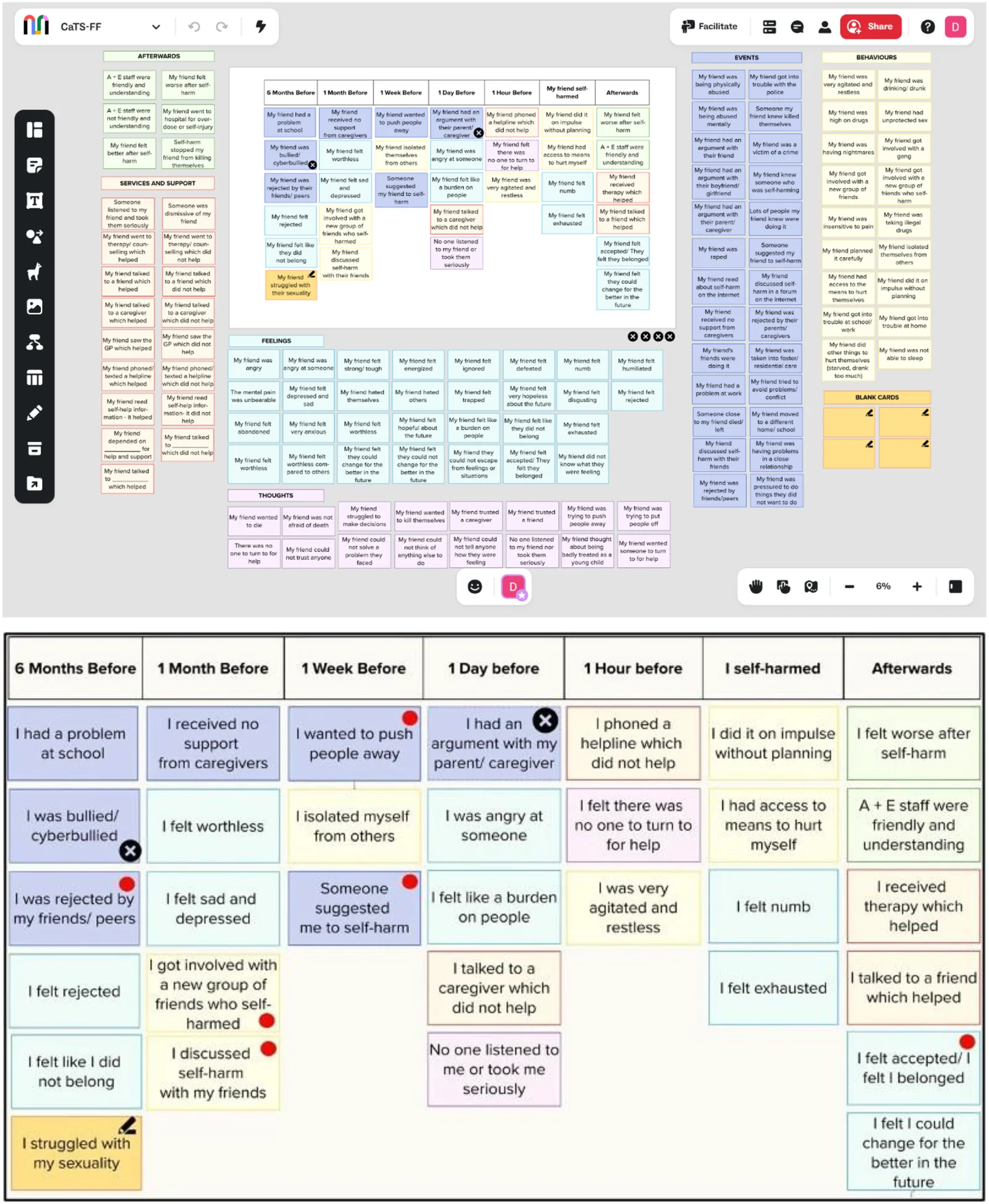
Mural board example for CaTS-FF

The delivery of CaTS has been versatile, with participants successfully completing the task using physical cards [3, 47, 49, 56] or in online spaces with digital cards (private website / Mural / MIRO / ReactJS-based web-app, see Fig. 4) [52–54, 64]. Typically, researchers interacted with participants in face-to-face settings or through online calls, facilitating discussions around card choices and offering support. This interaction enables researchers to delve further into the depth of data provided by CaTS through cards alone, allowing them to query components or thought processes as participants progress through the task.

However, in-person research activities can be time-consuming, limit recruitment opportunities, and act as a barrier to openly sharing experiences [67]. CaTS has been delivered remotely [50; 51], CaTS-parents [55, 64]), through the use of posted physical cards, third-party collaborative online whiteboard platforms (Mural/MIRO) and is specifically being developed as a digital tool (CaTS-Digital, CaTS-D [54]; CaTS-App). This allows for greater flexibility and convenience and acts as a resource-effective tool for researchers (i.e., cost and time). Digital collection also supports recording of *how* CaTS is completed and offers further functionality as – without the space-constraints of a table-top – cards can be repeated and placed multiple times throughout a timeline. As many of these studies took place during the COVID-19 pandemic, the digital transition was also necessary to mitigate ongoing environmental, health, and social policies that limited research practices. Largely sustaining this emphasis on exploring digital contexts parallels many research and healthcare practices since the COVID-19 outbreak.

Digital delivery of CaTS allows for independent completion. For some people, being able to complete the task in their own time, without any perceived pressure from the researcher’s presence, might be important. CaTS can be presented as a standalone research activity. Alternatively, the card sort might represent one stage of a wider research protocol. For example, Bilello et al. [51, 53] used CaTS as an initial activity to ground and structure participant’s perceptions and to establish a shared understanding between participants and the researcher regarding their/ their friends’ self-harm experience. Participants were then invited to discuss these perceptions in greater depth through interviews, using their completed CaTS as a prompt.

As with face-to-face work, it is important to consider ethics and the safeguarding of participants in a digital environment. An ever-growing body of research evidence indicates that research addressing self-harm and suicide is safe [68–70]. However, it remains important to be mindful that while at their core, they remain unchanged from many in-person contexts, safeguarding considerations might manifest differently in digital content [71, 72]. For example, VAS can be used pre- and post-task to assess for any impact of task completion on mood. While this might give valuable group-level information while working remotely, it might not be considered sufficient to meet ethics requirements on an individual basis, as no information is gleaned about the wellbeing of any participants who withdraw. There are many ways to address this, which are often an extension of established practice for in-person working. For example, careful consideration should be given to the embedding of signposting information to verified sources of support throughout protocols and formats, such that they are readily available to a participant should they withdraw from the study prior to completion. In our work adapting CaTS for autistic adults, we used a wellbeing plan developed in partnership with autistic people and those who support them – and building upon similar wellbeing plans co-developed by the National Institute for Health and Care Research (NIHR) Greater Manchester Patient Safety Research Collaboration (GM PSRC) and the See What I’m Saying? Project (https://seewhatimsayingproject.weebly.com/) - for use across sensitive studies (available from: https://sites.google.com/view/mentalhealthinautism/resources/tools). This wellbeing plan facilitates a discussion between researchers and participants as to how participants can be supported to take part in the study safely, including a plan if the participant becomes distressed or needs support.

In digital contexts, safety considerations should also extend to digital security and data security (especially when researching sensitive topics). Bird et al.’s [54], specific version of CaTS-D has been established as a usable and feasible delivery system, allowing for the potential of large-scale, safe, and secure data capture. This ability to safely scale the use of CaTS offers exciting new avenues for further analytic interpretations by including features such as allowing multiple card selections across timepoints. Having this curated electronic system ensures robust and reliable methodology, reducing the possibilities of human error (e.g., data inputting).

### Cards: content, context, and use

The CaTS is designed to be applied flexibly across differing definitions and experiences of self-harm. In many applications, this flexibility allows participants to define self-harm in ways that are meaningful to them, consistent with participant- and lived experience-led approaches [73]. Alternatively, CaTS can be used within more focused, researcher- or project-defined parameters where required (e.g. [48, 50, 54, 65]). This adaptability enables CaTS to be used across a wide range of conceptual frameworks and experiential contexts, supporting its utility in diverse self-harm research settings. Furthermore, by adjusting the definition adopted (e.g., self-harm versus non-suicidal self-injury), CaTS offers the opportunity to compare trajectories and to identify key similarities and differences.

Since CaTS’ inception in 2016, factors have emerged which need inclusion to ensure the tools’ relevance and appropriateness for contemporary assessment. For example, concerns regarding the impact of social media [74] or impactful global events (e.g., COVID-19 [75]); have been documented in the literature and could warrant inclusion with CaTS. CaTS retains relevance due to the ability for the card deck to be updated depending on both environmental and personal contexts CaTS is being used in and with. As an example, Bird and colleagues [54] have adapted CaTS with and for LGBTQIA+ populations leading to additions and changes to the card deck including the inclusion of 13 new cards (including an additional time point *“before 6 months”*). These additions captured specific experiences faced by the LGBTQIA+ population (e.g., “*I was questioning my sexuality or gender identity*”) but also more general factors which might be relevant to wider populations (e.g., “*Self-harm gave me a feeling of control*”). Similarly, in CaTS-FF, social factors which are necessary to convey components of perceptions of friends’ self-harm and relational dynamics were included within the cards (e.g., “*My friend wanted to push people away*”, “*Someone suggested my friend to self-harm*” [51]). Pelton et al. [55] collaborated with an autistic advisory group to review the appropriateness of CaTS for autistic people. As with the LGBTIA+ study [54], here the timeline was extended to include “longer than 6 months” to account for relevant earlier life experiences related to self-harm. Autistic participants who then completed CaTS also contributed new cards reflecting experiences such as unmet autism and mental health support needs, and poor understanding of or poor support for autism (e.g., “*I had a diagnosis and couldn’t get my head round it*”, “*My workplace did not understand me*”, “*No therapy or support was offered*”). Most of these cards were unique to individuals and did not meet the frequency threshold to be analysed further. Given the high number of blank cards used by autistic adult within this study (145 unique cards across 29 participants), the blank cards warrant further exploration, both in terms of exploring any common themes and to establish whether additional cards should be added for this group.

Modifications to CaTS for use with young people have included expanding the card deck to include multiple constructs of ‘impulsivity’ to allow for a more nuanced, scaffolded exploration of impulsive behaviour as an important transition to self-harm [49]. Following PPI recommendation, the original timeline has also been extended to include earlier ‘over 6 months before’ and subsequent time points ‘immediately afterwards’ and ‘later on after’ [50]. Given evidence that young people who self-harm often act within 10 minutes of first ideation [76], the timeline has also been compressed to start at ‘one day before’ and include the minutes up to an episode of self-harm [49]. Following these adaptations, the original CaTS’ timeline has been updated to include a more fine-grained time frame which explores the hours and minutes leading up to a self-harm episode.

Together, these evidence-based adaptations highlight the social and cultural competency CaTS offers as a flexible methodological approach. Further adaptation should continue to incorporate changing social views and our evolving understanding of self-harm as well as refining the phrasing of cards within the card deck.

CaTS is a person-centred approach to understanding self-harm and has traditionally been used to present one’s own narrative leading to an episode of self-harm [3, 47, 49, 50]. However, CaTS can be used to describe a perceived self-harm trajectory from an outside party, for example, a friend or parent [51, 52, 54]; the card sort can be self- or other- oriented as a task. Furthermore, to support those discussing others’ self-harm, hypothetical vignettes using CaTS can be offered to aid understanding of self-harm and influential experiences.

Given a starting point of 117 cards (with the option to include novel cards via the additional ‘blank cards’) in the original version, and an expanding card deck and timeline as detailed, researchers may need to be mindful of how many cards are being presented to participants to alleviate feelings of overwhelm. Of note, in involvement work, young people and autistic adults have suggested that a large deck of items is important given the complexity of the behaviour and the validation this brings that someone else may have experienced this in the journey toward their own self-harm. On the other hand, support may need to be provided to help manage the deck. Support, in this context, could be a researcher facilitating the task or being available to offer guidance and address any questions. We hold that it is also helpful to consider support more broadly, for example considering the information a participant receives prior to engagement. This could include having clear instructions sent in advance of meeting with the researchers. This might include written instructions for familiarisation, a short video demonstration to introduce the task, and examples of CaTS’ cards. Factors such as having cards in categories and colour-coded have also been reported as supportive. Bird et al. [54] have found that when participants complete CaTS-D independently, cards need to be concise and direct to maintain engagement. While different permutations of CaTS will be most suitable to individual research studies and populations, clear explanations as to choices, decisions, and analytic processes are recommended to provide clarity, comprehension, and integrity of the research.

## Interpretation

To allow analysis of multiple cards selected across timepoints, research using CaTS in-person utilises lag sequence analysis (see 39). This methodology involves identifying the frequency at which cards occur. Townsend and colleagues [3] identified low, medium, and high-frequency cards by observing distinct changes in the gradient of a diagram similar to a scree plot. To ensure data is manageable, it is necessary to limit the number of items in the sequence analysis. Therefore, only high-frequency items are included. Once high-frequency items are identified, lag sequential analysis determines the frequency of every potential two-card sequence (antecedent-sequitur pairing). For example, how frequently does “*I felt very anxious*” occur after “*I had an argument with my parent/caregiver*”? Pairing frequencies are tabulated in a transition two-way frequency matrix: antecedents and sequitur cards in rows and columns respectively. Chi-square analysis is performed to test cross-dependency and standardised normal residuals (SNR) to identify which card pairings are more strongly aligned to the sequential structure than others. Transitions are important when their SNR achieves a value indicating significance. Authors are required to make a choice about the value threshold [77]. Here, a criterion value of >2.0 was selected to ensure important transitions could be easily identified in a state transition diagram [3]. State transition diagrams include arrows which are demonstrative of links between items which reached the criterion value. This indicates the two factors are sequentially related. Using the aforementioned example, ‘I felt very anxious’ followed ‘I had an argument with my parent/caregiver’ more frequently than would be expected if the data contained no sequential patterns. Items with stronger links between them are identified by wider arrows. Occasionally, three-item sequences are identified, however large data sets are required to examine the order of these relationships [3].

A limitation of the original CaTS work was that participants could only use each card once within their sort. as this was required to perform lag sequential analysis. Subsequent iterations of CaTS have addressed this but, therefore, require a different analytical approach. Lockwood and colleagues [50] utilised Indicator Wave Analysis (IWA [78]), which allows multiple, simultaneous, and sequential events to be analysed across multiple time points. IWA produces easily interpretable diagrams (called indicator wave diagrams) that profile factors (indicators) prevalent to other factors at a specific time point). Analysis of individual cards is too complex for IWA, consequently, items are reduced by grouping cards into themes or categories. In Lockwood and colleagues’ study [50], this resulted in the development of 17 categories. Once categories are identified, items are organised into timepoints along the timeline and item frequencies are calculated at each timepoint. Frequencies are put into tabular format detailing the number of occurrences of cards within categories across the timeline. Finally, a chi-square analysis is performed to produce standardised residuals (SRs), which provide a measure of the strength of differences between observed and expected values. Employing this analytical methodology allows researchers to identify categories occurring more frequently than would be expected by chance. IWA also identifies associations between categories being selected at a specific time point as being more likely to occur than by chance. The IWA does not provide a prediction of behaviour, but rather a “characteristic signature” visible at time points in relation to self-harm.

CaTS scaffolds qualitative enquiry which facilitates contemplation and discussion about complex constructs and behaviours and can capture and distil the way that self-harm is experienced and understood at an individual level [49, 53, 55]. CaTS permits individuals to construct a personal representation of their unique experience. As a methodology, it provides a structured springboard for thinking, reflection and dialogue. This might be especially critical in qualitative work with young people, as well as in populations for whom the processing and describing of emotions and responses may also still be developing. Crucially, the process of pinpointing and describing salient factors is participant-led which helps to reduce power differentials between researcher and participant. As such, this helps maintain a balance between participants sharing their stories in their own terms and the researcher’s ability to explore key research questions by collaboratively unpacking specific cards. Furthermore, CaTS is suitable for qualitative analysis. In two interlinked studies, Bilello and colleagues [51, 53] used semi-structured interviews to explore the experiences of young adults who self-harmed or who had a friend who self-harmed. Interviews were prompted by adapted versions of CaTS (CaTS-FF; CaTS-AF), whereby cards contained friendship-related factors, in line with the primary aims and research questions. Thematic analysis was used to explore common and unique factors across participants narrative’s while cards supported the identification of shared salient experiences (e.g., the role of isolation [53]);

## Beyond research

### Training, education and capacity building

Effective training is a critical pillar in increasing the timely provision of meaningful and appropriate responses, as well as evidence-based, psychologically-informed approaches to supporting people (of all ages) who self-harm. Effective training facilitates capacity building by increasing the working knowledge base; enhancing both awareness and access to accurate information. Training also holds the potential to upskill respondents by building confidence in having conversations that might traditionally be considered ‘difficult’. From the start, out involvement work has highlighted that training staff is a priority in the dissemination of CaTS. For example, in the Listen Up! Project [3] the YPAG saw sharing findings and providing training to staff in schools, social work and care workers as critical in helping young people to get the support that they need sooner.

Consequently, the application of CaTS has been expanded to explore its relevance and acceptance as a training tool, across clinical and non-clinical contexts. CaTS has been used as a visual framework and adjunct to existing training modalities. Understanding factors associated with self-harm, the complex interplay of these factors in the pathway to self-harm, and the identification of relevant points for intervention is aligned with priorities outlined by key competency frameworks in this area. For example, in the UK, the Self-harm and Suicide Prevention Competence Framework (https://www.ucl.ac.uk/pals/research/clinical-educational-and-health-psychology/research-groups/competence-frameworks/self-harm) outlines competencies around ‘knowledge of issues related to self-harm and suicide prevention’, as well as ‘assessment, formulation, engagement and planning competencies’.

In our work, we have used CaTS in training with Child and Adolescent Mental Health Services (CAMHS) specialist (tier 3) self-harm service staff team members. Here, staff members attending the training were presented with the card sort and, as groups, aimed to complete a timeline exploring what might be happening in the life of a young person accessing their service for assessment and intervention. The training emphasis centred on exploring and understanding the complexities that might surround self-harm. CaTS can also be used as a workshop tool. Here, the training objectives centred around identifying points of intervention. To facilitate discussion, we presented a completed (hypothetical) card sort as a way to communicate a presenting case for discussion. Trainees could also be presented with a (hypothetical) case vignette and complete a sort based on this information, exploring temporal dynamics within the pathway. CaTS represents a way to synthesise complex biographical information in a readily accessible format. Training feedback to date has been positive; formal assessment of the effectiveness of including CaTS in training provision represents a valuable future avenue for research.

The scope for CaTS as a training tool extends beyond the traditional audiences of competency frameworks, where reference is typically for a given professional group. CaTS can also be readily applied to education programmes – including education-based intervention development - with informal supporters, for example, with friends and family members of people who self-harm. To date, education-focused work has explored parents’ understanding of their child’s self-harm (CaTS-parents). Broadly, this group-based work has explored: (1) understanding young people’s experiences of self-harm (motivations); (2) exploring de-escalation and coping; (3) having conversations about self-harm; (4) the importance of family dynamics. Future work would seek to explore developing and evidencing the acceptability, feasibility and effectiveness of an education-based intervention for parents and carers. The inclusion of informal supporters is paramount in the promotion of comprehensive support, not least because help seeking often falls outside of professional services, particularly first disclosures of self-harm [79].

### Clinical practice

Much of our evidence-building to date has focused on CaTS as a research tool. However, from the start, involved young people, participants and clinical colleagues have provided consistent feedback that they would also like to see CaTS developed as a clinical tool and/ or an app. Within a clinical context, CaTS could be applied to the process of assessment and formulation, as well as intervention planning; identifying significant antecedent-sequitur transitions in the pathway to self-harm could help identify and prioritise targets for intervention and develop tailored and effective approaches [27, 29]. Participants in the original CaTS study reported ‘light bulb’ moments where they felt that they had gained new understanding of their self-harm by completing the task [3].

As a tool, CaTS also holds utility in rapport building and could assist in developing a sense of safety within a therapeutic context. As such, CaTS could represent a novel therapeutic assessment; when done collaboratively, structured psychological and psychosocial assessment can not only inform treatment but in itself be a purposeful therapeutic tool, producing meaningful therapeutic change. When used as an adjunct to therapy, therapeutic assessment can be used to explore questions, reinforce insight and tailor an approach based on shared phenomenological understanding [80–84]. Recent work exploring research participant’s experience of completing CaTS for suicide attempts and non-suicidal self-injury in both adolescents and young adults shows perceived feasibility and helpfulness of using CaTS in therapy [65]. NHS England’s current transformational programme – Culture of Care (https://www.rcpsych.ac.uk/improving-care/nccmh/culture-of-care-programme) – emphasises the importance of personalised approaches to risk assessment and patient safety, embedded within trauma-informed, autism-informed, anti-racist, relational mental health care. CaTS aligns well with these fundamental principles, offering a novel approach to undertake collaborative assessment and a tool with which an individual can explore their self-harm journey meaningfully and on their own terms.

A clinically adapted CaTS could be completed alongside a therapist (side-by-side) and explored collaboratively. The card sort is interactive and physically engaging in a way that many traditional question-based assessment tools are not. In our research work, autistic participants spontaneously reported that the CaTS helped them understand their own journey into self-harm and asked for a copy of their completed CaTS to share and discuss with the person who supports them [55]. This alternative and/or adjunct approach might be particularly important if an individual has already gone through multiple assessment processes in their lifetime, to mitigate and manage potentially difficult feelings that they have explained these things before. Clinicians are keen to explore CaTS in their practise; To date, as a research team we have been approached to discuss the application of CaTS in CAMHS, adult inpatient and community mental health, forensic services and autism diagnostic services. We have also discussed the role of CaTS delivered within education-based healthcare provision e.g. via school nurses or through CAMHS-based mental health support services (both NHS and third sector) teams.

CaTS does not necessitate verbal communication for full completion of the tool; clients could talk as much or as little as they want as they work on the task. By contrast, most extant assessment processes and tools rely on verbal responding and struggle to adapt to scenarios where someone may not want to, or may not be able to, communicate verbally. This flexibility may be important to meet sensory or processing needs, access requirements and reasonable adjustments (e.g., in the context of anxiety or neurodevelopmental conditions), as well as contributing more broadly to approaches that aim to meet the client where they are at. Disclosure can be difficult, especially within the context of a new therapeutic relationship. By exploring timelines with CaTS, a client would be able to identify key thoughts, feelings, events and behaviours without saying or naming them. CaTS offers an opportunity for a client to – in the first instance - acknowledge a card as pertinent in their journey without further exploration at that time. Within the CaTS framework, this is easy to flag by adding a sticker to a card [51]. CaTS also offers a useful timelining tool which may act as a framework to ‘zoom in’ and ‘zoom out’ temporally to explore proximal and distal factors, and to think about intervention at different points. It can also serve as a visual tool to acknowledge the complex interplay of factors and just how much might be going on in the life of the client, aiding compassionate responding.

We believe that CaTS’ flexibility offers further potential for exploration - and evaluation - in clinical practice. Traditionally, CaTS explores factors over a 6-month timeframe. However, this frame of reference can be manipulated to best suit the clinical focus. Similarly, the reference episode could be the last incident of self-harm. Equally, the reference focus can be modified to the first episode, any given episode, or an instance in which the participant had thoughts of harming but did not hurt themselves (see 52 and 58 for examples). These pathways can then be explored comparatively to uncover similarities and differences. CaTS could be used at assessment or intake - captured in a photograph - and then revisited throughout a clinical intervention to see if cards need to be added or amended as understanding changes. Currently, CaTS instructions emphasise selecting the thoughts, feelings, events and behaviours that the person believes are relevant in their journey to self-harm. Arguably this requires insight to select and understand the potential impact of distal factors. There is potential to explore different variants of the tool’s instructions. For example, you could first ask someone to select cards that are relevant to them across the timeline and subsequently ask about perceived relevance to self-harm experience(s).

As part of an UKRI funded programme of research (Digital Youth), work is currently underway to co-develop and test a web-based app version of CaTS (CaTS-App) delivered on a tablet or screen. CaTS-App is being developed primarily as a clinical rather than a research tool, to be used collaboratively by a young person and practitioner side-by-side during clinical sessions to support improved understanding, communication and assessment of self-harm. CaTS-App has been developed in response to academic, clinician and young person feedback and builds on recommendations in NHS guidelines [44] which underline the need for assessment and intervention tools to support shared understanding and collaborative decision-making around self-harm needs and support.

Following extensive co-production and stakeholder engagement work, and building on the body of CaTS research to date, adaptations have been included in the prototype version of CaTS-App. These include a flexible timeline which can be modified as required or adapted to include notes and narrative markers (e.g. ‘start of term’ or ‘Christmas’) in addition to chronological timepoints; an expanded deck of cards and categories co-produced with stakeholders to include contemporary risk factors (such as cyberbullying) with the ability again to add individual notes and context to each card selected, and the option to include strengths, attributes, and values-based factors as part of client pathways. There are also recommendations for session structure and manuals and video materials to support the delivery of the tool in sessions. As a web-app tool CaTS-App draws on the affordances of digital innovation, including additional functionality in card selection, recording and reporting processes, usability features and personalisation. Exploratory work for the potential fit, feasibility and implementation of a practitioner-supported digital version of CaTS within frontline settings suggests there is strong appetite among practitioners within healthcare, education, and third sector settings [44, 85].

### Public engagement and understanding of self-harm

Public understanding of self-harm is important. Self-harm is prevalent, yet often misunderstood. While public responses to self-harm can be helpful and compassionate, fear and stigma may limit this [86–88]. Involvement and engagement are paramount in research to ensure the acceptability, applicability, importance and appropriateness of research questions, methods and approaches [44]. Involvement and engagement work can also meaningfully positively impact dissemination - including via non-traditional methods - and research impact. To date, we have used CaTS in an engagement context to explore public understanding and as a scaffolding tool for conversations and prompting questions. For example, we have taken CaTS to Café Connect, an innovative project - based on citizen science approaches - to engage with the public on health-related topics via a reciprocal process, within an existing community setting (https://osf.io/preprints/psyarxiv/28sne_v1). Café Connect included involvement and engagement activities across the lifecycle of research. Specific focus groups were held to explore adapting CaTS for populations with high unmet needs (e.g., those experiencing disordered eating and self-harm) through to the general public [85]. CaTS can be used successfully as an involvement and engagement tool in both private and public spaces, demonstrated by members of the general public being happy to engage with CaTS. CaTS in this context led to evidence-based discussions and ‘myth busting’ and elicited useful insights and novel understanding of lived experience priorities and future research directions. The tool has versatile application in an engagement context and can be used to think hypothetically, based on a vignette, or specifically (with a self or other reference). This is an important avenue for CaTS given that planning safe, responsible, engaging and interactive public engagement work directly addressing self-harm and suicide can be challenging.

## Future directions

### Research

Currently, research is underway to extend the application and reach of CaTS. The aforementioned digital version of CaTS, CaTS-App, is moving towards acceptability and usability testing within clinician/young person dyads after going through multiple iterative and collaborative design phases with young people and practitioners. Combining structured and systematic involvement with research has, to date, focused on design, acceptability, usability, and impact. This work has identified important new avenues of expansion, including a recommendation for including more protective and positive contributing factors as part of journey mapping. Across the research literature, our knowledge of protective factors is not well developed [89]. CaTS has begun to add in more protective cards but still work in progress, and this offers a valuable opportunity to further our understanding (as well as having potentially positive impacts on how CaTS is experienced). Completion of a card-sort on the CaTS-App includes an opportunity to include personal attributes, skills and values, adopting a more strengths-based approach than previous versions of the task. The aim is to support young people to recognise and identify personal reserves and opportunities which may help them to make choices other than self-harm. This is also an important opportunity for rapport building. Given the scarcity of evidence on protective factors, it is likely that work here will need iterative refinement. Moving forward, research to pilot CaTS-App will be a critical focus, considering acceptability, feasibility and efficacy. Economical evaluations are also important, as well as thorough exploration of potential mechanisms of implementation, including conversations with digital companies.

CaTS has demonstrated considerable strength in its flexibility as a research tool. The task is readily adaptable to reflect population specific, construct-specific and culturally-specific factors. While we have been approached for permission to translate CaTS for international use, we are not aware of any teams that have published work using a translated CaTS. An important avenue for future work would, therefore, be to seek to understand both the utility of CaTS outside of ‘WEIRD’ (Western, Educated, Industrialized, Rich, and Democratic) country contexts and to compare the resultant card sorts cross-culturally. Furthermore, CaTS is currently text-based. Future research may wish to explore whether simplified versions, graphics-based, or picture-supported card sorts might expand the populations with which CaTS can appropriately be used (for example, with young children or individuals with co-occurring intellectual disabilities, language and communication difficulties). As the reach of CaTS expands, it is likely that further amendments and additions to the card deck will be required. CaTS can fluidly accommodate this. As our understanding of self-harm evolves over time, the core CaTS deck can be updated. Alternatively, it may prove to be more appropriate to retain the current core card deck or develop and maintain group-specific decks (particularly if differing presentational or representational formats are used).

To date, CaTS has primarily focused on self-harm antecedents and sequiturs. While CaTS currently captures the more immediate ‘afterwards’ of self-harm, this area of the task could readily be expanded. Future work would benefit from exploring what happens after self-harm, and whether particular thoughts, feelings, events, behaviours or support and service responses increase or reduce the risk of subsequent self-harm. Such research would provide evidence of factors which may be important targets for post-self-harm interventions. CaTS has been used primarily to understand pathways to self-harm behaviour. Bilello [51] and del Carpio and colleagues [56] have begun to used CaTS to explore thinking but not acting (self-harm ideation in the absence of enactment), or instances where an alternative behaviour is enacted (an individual copes with a behaviour that is not self-harm). This represents an important avenue for future inquiry. Comparing pathways with different outcomes may offer novel insights in a way that is easily understood and visually evident. While conceptualised for self-harm, CaTS is flexible and would work for any index behaviour, event or episode. In early work, we have piloted exploring the card sort methodology to explore pathways leading up to a relationship conflict (as these are often events proximal to self-harm [79]). We have also begun to explore, through involvement work, the feasibility and acceptability of using CaTS in more complex presentations, for example, people experiencing difficulties with eating, as well as self-harm. Beyond self-harm, CaTS holds the potential to be amended to examine pathways to other maladaptive health outcomes (e.g., substance use) or behaviours (e.g., gambling).

Using CaTS emphasises the complexity of self-harm and the importance of not jumping to conclusions about what might be happening for someone. While many cards may be relevant in someone’s pathway to self-harm, this relevance might not be equally weighted. A future direction for the card sort would, therefore, be to explore the possibility of indicating the degree of relevance or the perceived relative importance of each card. This consideration has been included in the CaTS-App prototype where digital functionality allows for a value indication to be included on items selected. Clinically, this might naturally progress into considering intervention planning, goals and treatment targets. Finding the right words to explain an experience can be difficult. CaTS scaffolds this process and can make thinking about thinking more concrete. We could therefore hypothesise that CaTS supports mentalisation - the ability to understand one’s own and others’ mental states and consequently link these to intentions, actions and behaviours. However, to date, this - or indeed alternative possible mechanisms of action - has not been tested empirically.

The development of online versions of CaTS holds to potential to collate much larger data sets than has previously been achievable with in-person research. This offers new possibilities for exploring the data quantitively, for example exploring the use of Network Analysis, and identifying potential new unique proximal and distal risk and protective factors for self-harm not previously identified in under-researched high-risk groups (e.g. autistic people).

### Beyond research

CaTS supports the development of shared understanding and the identification of intervenable targets. These are essential components in assessment and formulation, intervention planning, the development of clinical interventions, and in self-harm policy making. We are continuing to develop CaTS-App, which is designed to be used in clinical settings to identify individual protective factors and key tipping points for service users to better understand their self-harm. Should the results of this work prove promising, it is essential to consider implementation and the practical and logistical barriers to disseminating knowledge and making it usable in practice. For an app, this is likely to involve collaboration with a technology company to provide rapid scalability. However, this means understanding matters of data security, intellectual property, licensing and industrial partnership working usually beyond the scope of self-harm and suicide prevention researchers. Moving forward, it is important that we are working towards building diverse and interdisciplinary teams, including those accustomed to working with industry partners. It is paramount that lived experience partnership remains centred in such development to ensure CaTS stays meaningful and useful to end users.

While CaTS is being developed clinically, not all front-line staff are clinicians and many first and early disclosures of self-harm are made to informal supporters [79]. As noted above, we have begun to explore how CaTS can be used as an adjunct to existing training provisions. Given the prevalence of self-harm – and the paucity of resourcing often encountered – it is important to think about how CaTS can most fully, yet safely, be shared. While intensive training would is not required to use CaTS successfully, moving forward, it would be important to develop flexible guidelines on how to use the tool. These may be practitioner guidelines – for clinicians or for use in schools – but equally could encompass peer-delivery methods. Exploring optimal training formats represents an important direction for future involvement work. It would be important to consider what ‘wrap-around’ materials and resources different people (e.g., clinicians, service providers, teachers, parents/caregivers, and informal supporters) feel that they would benefit from - in conjunction with having access to CaTS - and to explore possible training opportunities and extensions, for example, train-the-trainer style formats. Adaptation of CaTS to consider self and other-perspectives opens possibilities to use CaTS in novel dyadic contexts (beyond individual-clinician), both for research (for example, comparing perspectives of individuals who self-harm and supporters) but also in clinical practice (for example in family-based dialogic interventions or mentalisation-based family interventions).

### Forwards together: developing a global CaTS community

Given the wide-reaching applicability and utility of CaTS across key stakeholders, CaTS offers a real opportunity to build community and dialogue between groups often working towards the same goals but with little-to-no meaningful communication, co-production or opportunities for shared learning. Developing a connected network of those using - or interested in using - CaTS offers possibilities to foster two-way learning and to narrow implementation gaps. Bringing people together in discussion, while holding a central repository of resources pertaining to the tool, is also likely to elucidate novel applications of CaTS. We want to foster collaboration as well as feedback on CaTS. For example, the CaTS cards are updated over time. We have already seen how and when new cards have been identified, and it may be that over time some current cards seem less relevant. One feature of a CaTS-central network could be to define the timeline over which core cards are to be reviewed and the process by which this is achieved. Sustainability and inclusion are centrally aligned with the co-production and involvement principles core to CaTS.

**Table 1:** Summary table of all CaTS research to date

Study	CaTS version	Research question (s)	Population	Format	Facilitated by a researcher?	Timeline alterations	Cards (added or removed)	Reference for self-harm episode	PPIE involvement and brief outline	Key learning
Townsend et al. (2016) [3]	CaTS	To examine key patterns in antecedents to young people’s first and most recent episode of self-harm using the novel CaTS method	Young people aged 11–21 years old	Paper, in-person	Yes	n/a	n/a	Self-reported first and most recent self-harm episode	Self-harm research group (SHRG) advisory group, hosted by self-harm CIC Harmless. Regular advisory group meetings with three- to six young people with lived experience of self-harm (including some with experience of being looked-after). Activities around prioritising research plans and findings and selecting the card items. Input around dissemination and implications …	• CaTS revealed important transitions in the pathway to self-harm • Factors most proximal to self-harm are amenable to intervention • For recent episodes, hopelessness and suicidality emerged as significant factors First ever self-harm (but not most recent) was associated with feeling better afterward
Wadman et al. (2017) [47]	CaTS	To compare key patterns in factors leading to an episode of self-harm in looked-after young people and young people who have never been in care	Younge people aged 11–21 years old	Paper, in-person	Yes	n/a	n/a	Self-reported first and most recent self-harm episode	Self-harm research group (SHRG) advisory group, hosted by self-harm CIC Harmless. Regular advisory group meetings with three- to six young people with lived experience of self-harm (including some with experience of being looked-after). Activities around prioritising research plans and findings and selecting the card items. Input around dissemination and implications.	• Self-harm can change and evolve over time; assessments need to reflect this
										• Looked-after young people reported feeling better after first self-harm; fearlessness of death, access to means, and impulsivity were reported as key in recent self-harm • Underlying emotional distress, particularly depression and self-hatred were important in both first and most recent self-harm CaTS would have clinical utility as an assessment tool
Lockwood et al., (2020) [49]	CaTS	To explore (1): young people’s understanding and experience of self-harm in the context of impulsivity-related behaviour, and (2) the salience of impulsivity and the wider emotional / cognitive context as a proximal or distal influence on self-harm	Young people aged 16–22 years in Further Education college settings (*n* = 15) with lifetime self-harm history	A modified paper-based version, used in conjunction with a semi-structured interview; In-person	Yes	Compressed timeline from: *“one day before”*, *“1 hour before”*,	Modified version included 30 items from original CaTS, 8 items identified in a supplementary survey, 5 items associated with expectations associated with self-harm act	A specific time when they had good recollection of experience	Adaptations to CaTS were based on findings from survey responses from young people developed in conjunction with youth advisory panel in one-to-one sessions	• Rash response to heightened arousal and poor cognitive management is a common characteristic of self-harm in young people • CaTS supported nuanced discussion The introduction of a pre- *“All about me”* card sort task supported mentalisation and rapport
						*“30 mins before”*, *“10 mins before”*, *“‘less than 10 mins before”*, *“5 mins before”*, *“at the moment of self-harm”*, to *“afterwards”*				
Lockwood et al. (2023) [50]	CaTS	To compare the interplay of factors leading to self-harm in young people versus adults; to explore differences between first-ever and most recent self-ham for both groups; to analyse using a novel Indicator Wave Analysis (IWA) approach	Young people aged 18–25 years and adults with a lifetime self-harm history	Online version of CaTS	No	*“Over 6* *months before“*, *“6 months before“*, *“1 month before“*, *“1 week before“*, *“1 day before“*, *“1 hr before“*, *“just before self-harm“*, *“immediately afterwards“*, *and “later on after“*	Reduction of card items to 89 cards by grouping similar items into a total of 17 categories	Self-reported first and most recent self-harm episode	Five PPI events held through Café Connect - focus groups with young people with lived experience. Three young people worked with team as Participant Engagers on data collection, analysis, interpretation, and at dissemination events including Café Connect at Night led by the participant engagers	• CaTS revealed the common importance of impulsivity, isolation, loneliness and entrapment in self-harm pathways. Notably, burdensomeness marked for adults only • IWA could support early identification of warning signs
Lockwood et al., (2023) [50]	CaTS-App	CaTS-App Phase 1; Planning and Discovery - Findings of our work with professional stakeholders and youth	Front line professionals from healthcare, education, third sector and social care settings (*n* = 63)	Online Survey and/or Interview/ Focus Group	Yes	n/a	n/a	n/a	Six young people as part of Digital Youth programme acted as collaborators by: developing research design; facilitating sessions; supporting analysis of co-production work. 11 young people were involved in three co-production workshops influencing core design and delivery processes for CaTS-App. Young people also took part in an online showcase event where we explored and sense-checked findings and interpretations of workshops. 63 practitioners from health, social care, education, third sector and industry shared insights on feasibility, acceptability, safeguarding and implementation through surveys, workshops and focus groups	**Stakeholders valued CaTS-App for**: • Collaboration (structured framework to support conversations) • Youth Voice (co-development increased value and authenticity) • Digital Application (appealing, enhances engagement & value-added capacity) **Key value for young people:** • Communication & Autonomy • Access, Safety and Privacy Concerns • Offer a structured process to deliver the CaTS-App • Adjustments to the content of the CaTS task
Bilello et al. (2024) [51]	CaTS-FF	To describe and explore how friends of young people who self-harmed (1): perceived, experienced, and responded to adolescents’ self-harm; and (2) influenced and were influenced by the self-harm experience	Friends of young people who self-harmed during adolescence	Both: paper copy and online (Mural)	Yes	Timeline was changed to reflect other’s self-harm (e.g., *“My friend self-harmed”* rather than *‘I self-harmed”*)	18 new friendship-related cards; 19 cards were modified/ combined	Friend-oriented episode of self-harm i.e., experience of friend self-harm	No additional formal PPIE involvement was conducted for the study beyond the original CaTS study. Card adaptations were informed by the literature and consultation with self-harm experts within and outside the research team	CaTS flexibility to be adapted to a third-person perspective allowed to represent friends’ perceptions of adolescent self-harm, while also facilitating reflection on the impact of the experience on friend supporters through the scaffolding and prompting of semi-structured interviews.
Brausch et al. (2024) [65]	CaTS	To explore pathways to suicide attempts and non-suicidal self-injury using CaTS and examining networks of cards that are distal and proximal to self-harm	Adolescents (ages 12–17) in a crisis stabilization unit	Paper-based	Yes	Inclusion of *“more than 6 months before”*; *“I attempted suicide”* or *“I self-injured”* used in place of *“I self-harmed”*	Cards adapted for American participants (e.g., ER, primary care doctor, foster care”	Self-oriented; most recent suicide attempt or most recent self-injury episode (all occurred within the past 12 months)	Feedback gathered from all participants on the CaTS task and how it may be implemented in clinical settings	There was some overlap in high frequency cards selected for suicide attempts and NSSI, as well as unique cards. Networks emerged for cards that are distal to self-harm (1–6 months prior) and proximal (1 week to 1 hour prior)
Lockwood et al. (2024)	CaTS-App	CaTS-App Phase 2; App Development – Findings from co-production workshops and professional stakeholders	Young people aged 18–25 years with experience of self-harm (*n* = 7)	3 online co-production workshops	Yes	n/a	n/a	n/a	Six young people as part of Digital Youth programme acted as collaborators, in developing the research design, facilitators of sessions, and supported analysis of co-production work. Seven young people took part in three co-production workshops influencing the navigation, messaging, personalisation, acceptability, safety, card, and timeline updates, resulting in an early working prototype; - Practitioners were asked to provide feedback on the planned prototype via online showcases	**Core priorities for the CaTS-App development:** • Safe (start from a point of safety and revisit throughout) • Sort (Deliver a card sort process including options for familiarisation and personalisation) • Strong (incorporate strength and hope factors into the app) • Next Steps (include interaction to support clarity and decision making)
Bilello et al. (2025) [53]	CaTS-AF	To explore friendships and self-harm in adolescence, specifically: a) To describe individuals’ perceptions of the meaning, role, importance and impact of friends on their experiences of self-harm over time and b) to explore how friendships and self-harm evolved and co-evolved over time.	Young people who self-harmed during adolescence	Both: paper copy and online (Mural)	Yes	No	Addition of 18 friendship-related cards; 19 cards were modified/ combined	First episode (self-harm onset) and most recent self-harm	No additional formal PPIE involvement was conducted for the present study beyond the original CaTS study. Card adaptations were informed by the literature and consultation with self-harm experts within and outside the research team.	• Use of CaTS in a qualitative context supported a shared understanding of participants’ self-harm experiences and enabled in-depth exploration of key research questions. It also facilitated the development of qualitative themes and the identification of common factors experiences shared across participants
Lockwood et al. (in prep) [52]	CaTS-parent	To explore CaTS as a potential tool to support parents to (1): explore and understand the complex and changing nature of self-harm in a young person, and (2) have informed, safe conversations about self-harm with others (e.g. with child, family, professionals)	Parents and caregivers	Online (miro)	Yes	Inclusion of *“longer than 6 months”*, *“1 hour before”*, *“immediately before”*, *“immediately after”*, *“later on”*	n/a	Experience of child’s self-harm or vignette example of self-harm	The study draws on prior PPI work which shaped the study approach and development of potential parent-facing tool. This included: online participatory workshops and offline tasks (*n* = 3 parents); interviews or focus groups (*n* = 12); user-testing workshop (*n* = 6 peer-carer peer support workers).	• Parents valued CaTS for improved understanding (psychoeducation), scaffolded communication, trusted connection, and space to practice and process
Brausch et al. (in prep) [48]		To explore pathways to suicide attempts and non-suicidal self-injury using CaTS and gather feedback on the potential of CaTS as a therapeutic tool	Young adults (ages 18–25)	Paper-based	Yes	Inclusion of *“more than 6 months before”; “I attempted suicide”* or *“I self-injured”* used in place of *“I self-harmed”*	Cards adapted for American participants (e.g., ER, primary care doctor, foster care”	Self-oriented; most recent suicide attempt or most recent self-injury episode (all occurred within at least the past 12 months)	Feedback gathered from all participants on the CaTS task and how it may be implemented in clinical settings	• There was some overlap in high frequency cards selected for suicide attempts and NSSI, as well as unique cards. Participant feedback was positive about the task and its potential as a clinical tool
del Carpio et al. (in prep) [56]	CaTS	To explore the factors that contribute to first episode and recent self-harm thoughts or actions among a sample of young people bereaved by the death of someone important to them	Young people in Scotland, aged 16–18 years	Paper-based	Yes	No	Blank cards provided, and participants added: [1] *“I thought I can’t do it”* [2] *“Bullied but they didn’t realise”*	Self-reported first- and most-recent episode	No additional formal PPIE involvement was conducted for the study. Participantsengaged in CaTS as part of a mixed-methods study involving interviews; study did not involve co-creation of the CaTS	• CaTS provided a structured yet flexible way for young people to engage with the topic of self-harm, and having a facilitator nearby allowed for explanations or reflections when desired • Colour-coding of the cards (e.g., into thoughts, feelings, events, etc.) helped participants navigate through the large deck more easily • A small sample size limited the ability to conduct the formal statistical analysis as planned
Pelton et al. (in prep) [55]	CaTS-A	To (1): adapt CaTS with autistic people, ensuring it is clear and accessible; and (2) undertake a pilot study to identify proximal, distal contributing factors, antecedents and consequences of self-harm in autistic adults.	Autistic adults	Online (MIRO)	Yes	Inclusion of *“longer than 6 months”*	n/a	Self-orientated, most recent episode	• a lived experience advisory panel (LEAP) of five autistic adults with lifetime experience of self-harm, were recruited from the Autistica Discover Network – a research charity maintained volunteers database. The LEAP met with the researchers six times over the course of the study, advising on acceptability of ethical safeguards, any adaptations to the CaTS instructions, editing/deletion/addition of CaTS cards, how to adapt the CaTS to an online task, and recruitment.	• Most commonly selected cards were *“The mental pain was unbearable”*, and *“I felt depressed and sad”*; a wide range of traumatic life events were distal to self-harm: agitation and restlessness and acting on impulse preceded self-harm; and feeling better and worse, exhausted and hopeless followed self-harm
Bird et al. (2025) [54]	CaTS-D	To capture qualitative views from people with lived self-harm experience including whether amendments or additions to CaTS are necessary to enhance the relevance and meaningfulness of the cards, particularly in LGBTQIA+ populations. 2) To pilot test newly-developed CaTS web-app (CaTS-D) to ensure acceptability 3) To explore self-harm pathways in general and LGBTQIA+ populations. Identify key group-specific antecedents which present important intervenable targets. Also, to examine each groups self-harm pathway using the framework of the Integrate-Motivational Model of Suicidal Behaviour[2]	General and LGBTQIA+ populations, aged 18+	Study 1) Face-to-face online; Studies 2 & 3: Online	Study 1: Yes Studies 2 & 3: No	See results	See results	Most recent	• A qualitative study with people with lived/living experience of self-harm to develop meaningful, relevant updates to CaTS/CaTS-D.	**Study 1:** • 12 new cards added, including a ‘Before 6-months’ timeline card, important to capture historic events impacting self-harm Neutralise gendered phrasing (e.g., changing ‘boyfriend/girlfriend’ ro ‘partner’) **Study 2:** • Pilot testing of CaTS-D showed it is a useable, acceptable research tool, which is easy to use and apply remotely with minimal instructions **Study 3:** • Cis-hetero and LGBTQIA+ pathways followed a similar pattern • Findings largely support the IMV model • Life events act as distal factors. Cis-hetero people may experience negative life events for a longer period • For both groups, insomnia, humiliation, defeat, loneliness, entrapment, and isolation act as Motivational factors in the weeks preceding self-harm, suggesting these factors are important in the transition towards ideation Impulsivity, planning self-harm, hopelessness, and executive function issues are salient proximally to self-harm for both groups • Acquired capability ony salient for LGBTQIA+ people proximal to self-harm • For cis-hetero people, negative social media peaked a month before self-harm suggesting problematic social media use should be discussed during intervention • Post-self-harm, both groups reported feelings of control, highlighting the potentially cathartic effects of self-harm
McDonald et al. (in prep) [55]	Used CaTS principles and methodology	To (1) understand the experiences of UK patients attending audiology who have tinnitus and may or may not have had suicidal thoughts and behaviours (SIBs) (2), develop a transdiagnostic model of tinnitus and SIBs informed by key theories and models of SIBs and participants’ lived experience, identifying key variables and transdiagnostic processes.	UK adults with tinnitus attending audiology	Face-to-face & online	Yes	We developed and presented set of cards which reflect tinnitus and suicide factors and which people reflected on in relation firstly to tinnitus and secondly, to suicidal thoughts and behaviours. There are cards for thoughts, beliefs, emotional and physical feelings, interpersonal /relationships, impact and contextual social factors, how I cope (blank) and who helps me to cope (blank). We invited participants to reflect on the sequence and process in experiences, and interactions between factors and elements. Participants talked through overall timeline from first experiences to the present, and within that timeline, most focussed on crisis points and gave a sequential account, the story, of the factors and interactions between factors which were key to these experiences.			Initial project design and inclusivity was discussed and refined with members of UoN/BRC Hearing PPI group, and the combined Mental health and PPI group. Helpful guidance given by PPI group on support and safety during card sort and interview process. The card set was reviewed and tested by two people with experience of tinnitus and suicidal thoughts and behaviours.	• CaTS principles were adapted to focus on SIBs and tinnitus • Thoughts of suicide can function as a (misdirected) coping strategy for tinnitus.
Babbage et al. (2024) [44]	CaTS-App	How to co-develop a digital CaTS tool using participatory (PPIE) methods; what processes enable safe, ethical, and meaningful co-production in self-harm research/tool development	Young people aged 17–24 with lived experience of self-harm (plus stakeholders incl. professionals) [psycnet.apa.org]	Mixed methods participatory co-design (workshops, interviews, surveys; online + face-to-face)	Yes	Workshops included reflections from PPIE on how they felt about the timeline and card content of the CaTS.			Extensive PPIE throughout: co-production with young people across ~48 months with involvement in online and face-to-face meetings to influence design decisions, data interpretation, and dissemination	• Digital mental health interventions for self-harm co-developed with young people with lived experience may lead to better outcomes, but young people are often excluded from involvement opportunities • This offers a case-study and working methodology for meaningful, safe, and responsible involvement in the early Planning and Discovery phase of the development of the CaTS-App • Activities to deliver involvement are in line with the five key priorities, drawing from existing and emerging models and principles from the fields of User Centred Design (UCD), Human Computer Interaction (HCI) and Responsible Research and Innovation (RRI). This paper contributes to important discussions about the process and value of centring and evaluating participatory processes in the development of digital mental health interventions for self-harm

* Solomes G, Wadman R and Townsend E. (In prep) [85]. Investigating perceptions of self-harm using the Card-Sort for Self-harm (CaTS): a content analysis. Unavailable for inclusion within summary table

## Conclusion

Taken together, CaTS has demonstrated acceptability and utility as a novel tool for the assessment and contextual understanding of self-harm. CaTS is sufficiently developed and evidenced to be used ‘off the shelf’ as a research tool. CaTS can also be adapted to suit specific contexts of populations. The process to adapt CaTS is explicated, and evidenced. The tool is versatile, evolving and – in addition to research – can be used safely and flexibly in diverse contexts, including in clinical practice, training and education and in public engagement.

## Data Availability

The article does not report primary data. No new datasets were generated or analysed in the preparation of this work. All information discussed in the article is derived from previously published studies, which are appropriately cited in text and in the reference section. The Card Sort Task for Self-Harm (CaTS) is available to access and download at https://selfharmresearchgroup.info/

## Abbreviations

WHO: World Health Organisation
LGBTQIA+: Sexual orientation and gender identity minorities
DBT: Dialectical Behaviour Therapy
TLFB-SA: Timeline Follow-Back Interview
PSAI: Pathway to Suicidal Action
EMA: Ecological Momentary Assessment
PPI: Patient and Public Involvement
YPAG: Youth Person’s Advisory Group
TFA: Theoretical Framework of Acceptability
VAS: Visual Analogue Scales
NIHR: National Institute for Health and Care Research
GM PSRC: Greater Manchester Patient Safety Research Collaboration
SNR: Standardised Normal Residuals
SR: Standardised Residuals
IWA: Indicator Wave Analysis
CAMHS: Child and Adolescent Mental Health Services
NHS: National Health Service
UKRI: United Kingdom Research and Innovation
WEIRD: Western, Educated, Industrialised, Rich, and Democratic
CaTS: Card Sort Task for Self-Harm
CaTS-FF: CaTS For Friends
CaTS-AF: CaTS About Friends
CaTS-A: CaTS Autism
CaTS-D: CaTS Digital
